# Role of independent versus chain pharmacies in providing pharmacy access: a nationwide, individual-level geographic information systems analysis

**DOI:** 10.1093/haschl/qxad003

**Published:** 2023-06-20

**Authors:** Inmaculada Hernandez, Shangbin Tang, Jasmine Morales, Nico Gabriel, Nimish Patel, Walter S Mathis, Jingchuan Guo, Lucas A Berenbrok

**Affiliations:** Division of Clinical Pharmacy, Skaggs School of Pharmacy and Pharmaceutical Sciences, University of California, San Diego, 9500 Gilman Dr, La Jolla, CA 92093, United States; Division of Clinical Pharmacy, Skaggs School of Pharmacy and Pharmaceutical Sciences, University of California, San Diego, 9500 Gilman Dr, La Jolla, CA 92093, United States; Division of Clinical Pharmacy, Skaggs School of Pharmacy and Pharmaceutical Sciences, University of California, San Diego, 9500 Gilman Dr, La Jolla, CA 92093, United States; Division of Clinical Pharmacy, Skaggs School of Pharmacy and Pharmaceutical Sciences, University of California, San Diego, 9500 Gilman Dr, La Jolla, CA 92093, United States; Division of Clinical Pharmacy, Skaggs School of Pharmacy and Pharmaceutical Sciences, University of California, San Diego, 9500 Gilman Dr, La Jolla, CA 92093, United States; Department of Psychiatry, Yale School of Medicine, 300 George Street New Haven, CT 06511, United States; Department of Pharmaceutical Outcomes and Policy, University of Florida College of Pharmacy, 1225 Center Drive, HPNP 2338, Gainesville, FL 32610, United States; Department of Pharmacy and Therapeutics, University of Pittsburgh School of Pharmacy, 3501 Terrace St, Pittsburgh, PA 15261, United States

**Keywords:** pharmacy access, pharmacy deserts, access to care, chain pharmacies, independent pharmacies

## Abstract

Pharmacy accessibility is critical for equity in medication access and is jeopardized by pharmacy closures, which disproportionately affect independent pharmacies. We conducted a geographic information systems analysis to quantify how many individuals across the United States do not have optimal pharmacy access or solely rely on independent pharmacies for access. We generated service areas of pharmacies using OpenStreetMap data. For each individual in a 30% random sample of the 2020 RTI International US Household Synthetic Population (*N* = 90 778 132), we defined optimal pharmacy access as having a driving distance to the closest pharmacy ≤2 miles in urban counties, ≤5 miles in suburban counties, and ≤10 miles in rural counties. Individuals were then categorized according to their access to chain and independent pharmacies. Five percent of the sample or ∼15.1 million individuals nationwide relied on independent pharmacies for optimal access. Individuals relying on independent pharmacies for optimal access were more likely to live in rural areas, be sixty-five years or older, and belong to low-income households. Another 19.5% of individuals in the sample did not have optimal pharmacy access, which corresponds to 59.0 million individuals nationwide. Our findings demonstrate that independent pharmacies play a critical role in ensuring equity in pharmacy access.

## Introduction

Pharmacy accessibility is crucial for access to prescription drugs. Over two-thirds of US adults use at least one medication, and 63% of prescriptions are filled at community pharmacies, rather than mail order.^1,2^ Beyond their role in medication dispensing, access to community pharmacies is of public health significance due to their established role in the administration of vaccines and rapid diagnostics, as demonstrated in the coronavirus disease 2019 (COVID-19) pandemic,^3^ and their expanding role in the provision of medication management services and preventive health screenings and services.^4,5^ Access to pharmacies is critical for equity in health care access because pharmacies are able to reach disadvantaged individuals who do not have access to other health care settings^4^ as well as rural residents with limited accessibility to primary care providers.^6^

Patient access to pharmacies and the services they provide is jeopardized by the rising trend of pharmacy closures,^7^ which have resulted in a net decrease in the total number of pharmacies.^8^ Pharmacy closures disproportionately affect independently owned pharmacies, which represent 38% of pharmacy locations across the United States but over 50% in Black and Latino metropolitan neighborhoods and over 75% in rural areas.^6,9^ These existing data on the distribution of pharmacy types across the United States are based on neighborhood-level analyses that do not allow for actual quantification of individuals who rely on independently owned pharmacies for access. Such information is relevant because independently owned pharmacies are poised to incur financial hardship as the vertical integration of insurers with pharmacy chains is increasingly excluding independently owned pharmacies from preferred networks.^10^

We performed an individual-level geographic information systems (GIS) analysis to quantify spatial access to pharmacy locations for a nationally representative sample of the US population. We determined how many individuals across the United States rely on independently owned pharmacies to access pharmacy services. We tested how reliance on independently owned pharmacies for access differed across racial/ethnic groups, income strata, and the rural–urban continuum. We further quantified the number of individuals nationwide who lack optimal pharmacy access.

## Methods

We obtained addresses for all open-door community pharmacies operating on July 1, 2020, from the National Council for Prescription Drug Programs.^11^ We restricted the sample to open-door pharmacies as they are open to the general public, as opposed to closed-door pharmacies, which may only provide medications to patients residing in certain facilities (ie, long-term care pharmacies, hospital pharmacies) or to individuals with certain affiliations (ie, Veterans Affairs’ pharmacies). We categorized pharmacies into chain or independently-owned pharmacies. The latter included independent and franchise pharmacies, which are independently owned pharmacies that have a franchise agreement.

We obtained from RTI International a 30% random sample of the 2020 US Household Synthetic Population, which was sampled at the block group level. The 2020 US Household Synthetic Population is a dataset composed of statistically accurate records for every household and person and therefore is representative of the US population.^12^ It includes information on age, income, race, and ethnicity, which is the product of matching high-resolution population distributions with the mix of households in each census block group.^12^ The RTI US Household Synthetic Population can therefore be interpreted as a dataset containing the census population of the United States without personal identifiers.

We computed pharmacy access measures using OpenStreetMap data and ArcGIS.^13^ Service areas were computed from driving distances for each pharmacy and were defined as driving distances ≤2 miles in urban counties, ≤5 miles in suburban counties, and ≤10 miles in rural counties. The 2- and 5-mile thresholds were selected based on the definition of the convenient access standard required for the pharmacy networks of Medicare Part D plans.^14^ In rural counties, we defined service areas as driving distances ≤10 miles based on the distribution of access to pharmacies across the sample (see supplementary exhibit A1). Counties were categorized as urban, suburban, or rural using estimates from the US Census Bureau for the proportion of rural population within a county.^15^ This variable has been used by the US Census Bureau to categorize counties based on urbanicity levels.^16^ Specifically, urban counties were defined as those with a <20% rural population, suburban counties as those with a 20%–50% rural population, and rural counties as those with a >50% rural population. We used this categorization rather than the US Department of Agriculture rural–urban continuum (RUCC) codes because RUCC codes classify rurality based on adjacency to a metropolitan area, which results in misclassification of rural counties adjacent to metropolitan areas as urban.^17^

We evaluated whether each individual in the synthetic population lived within the service area of an independent pharmacy and/or a chain pharmacy, and classified the population into three groups: (1) individuals with optimal access to chain pharmacies, defined as individuals who lived in the service area of a chain pharmacy, regardless of whether they also lived in the service area of an independent pharmacy; (2) individuals who relied on independently owned pharmacies for access, defined as individuals who lived in the service area of an independent pharmacy, but not in the service area of a chain pharmacy; and (3) individuals without optimal pharmacy access, defined as individuals who did not live in the service area of any pharmacy type. We extrapolated the results from the 30% random sample to the entire US population to estimate how many individuals lack optimal pharmacy access and how many rely on independently owned pharmacies for access.

We conducted chi-square tests to test how pharmacy access differed across subgroups defined by rurality, age, and household income. Because the distribution of racial/ethnic groups differs across the rural–urban continuum, we performed subgroup analyses by urbanicity to measure racial/ethnic disparities in pharmacy access. Finally, we mapped the distribution of individuals in each pharmacy access category across US counties.

## Results

The 30% random sample of the synthetic population included 90 778 132 individuals, of whom 51.2% were female and 63.5% were non-Hispanic White (supplementary appendix exhibit A2). We identified 61 715 open-door pharmacies, including 37 954 chains and 23 521 independently owned pharmacies.

Across the sample, 75.5% of individuals had optimal access to chain pharmacies (Table 1). These included 55.3% of individuals who had optimal access to both independently owned pharmacies and chains and 20.2% of individuals who had optimal access to chain pharmacies only (data not shown). Of the study sample, 5.0% had optimal access to independently owned pharmacies but not chains, which corresponds to 15.1 million individuals across the United States who rely on independently owned pharmacies for pharmacy access. The remaining 19.5% of individuals did not have optimal pharmacy access, which corresponds to 59.0 million individuals nationwide.

**Table 1.** Summary statistics for optimal access to pharmacies and projections for the US population.

Variable	Proportion of the sample (projected number across entire United States)		
	Population with optimal access to chain pharmacies^a,b^	Population with optimal access to independently owned pharmacies but not chains^a^	Population without optimal pharmacy access^a^
**Overall sample**	75.5% (228.5 M)	5.0% (15.1 M)	19.5% (59.0 M)
**Rurality^c^**			
Urban	80.3% (164.6 M)	2.6% (5.4 M)	17.1% (35.1 M)
Suburban	69.5% (40.5 M)	5.7% (3.3 M)	24.8% (14.5 M)
Rural	59.7% (23.4 M)	16.3% (6.4 M)	24.0% (9.4 M)
**Age**			
<65 years	75.9% (195.6 M)	4.9% (12.7 M)	19.2% (49.4 M)
65+ years	73.1% (32.8 M)	5.5% (2.5 M)	21.3% (9.6 M)
**Race/ethnicity**			
Non-Hispanic White	71.7% (137.8 M)	5.7% (11.0 M)	22.5% (43.3 M)
Hispanic	82.8% (39.9 M)	3.6% (1.7 M)	13.6% (6.6 M)
Non-Hispanic Black	81.6% (26.6 M)	4.6% (1.5 M)	13.8% (4.5 M)
Non-Hispanic Asian or Native Hawaiian	85.3% (12.8 M)	2.2% (0.33 M)	12.5% (1.9 M)
Indian Native	72.0% (3.1 M)	5.3% (0.23 M)	22.7% (1.0 M)
Other	79.3% (8.2 M)	3.9% (0.4 M)	16.7% (1.7 M)
**Household income**			
<$25 000	77.8% (43.8 M)	5.8% (3.3 M)	16.3% (9.2 M)
$25 000–$100 000	75.4% (116.1 M)	5.3% (8.2 M)	19.3% (29.7 M)
>$100 000	74.3% (68.6 M)	4.0% (3.7 M)	21.7% (20.1 M)

a Optimal access was defined as driving distance <2 miles in urban counties, <5 miles in suburban counties, and <10 miles in rural counties.

b Includes individuals who had optimal access to independently owned pharmacies and chains, as well as individuals who had optimal access to chain pharmacies only.

c Based on data from the US Census Bureau, urban counties were defined as those with <20% of rural population, suburban counties as those with 20%–50% rural population, and rural counties as those with >50% of rural population.

Rural residents were more likely to rely on independently owned pharmacies for access (16.3%), compared with suburban (5.7%) and urban (2.6%) residents (*P* < .001) (Table 1). Older adults and individuals with household income <$25 000 were also more likely to rely on independently owned pharmacies for access. Rural (24.0%) and suburban (24.8%) residents were more likely to lack optimal pharmacy access compared with urban residents (17.1%).

Across the entire sample, non-Hispanic White individuals were numerically more likely to rely on independently owned pharmacies for access (5.7%) compared with non-Hispanic Black individuals (4.6%). Subgroup analyses revealed that this finding was driven by suburban areas (supplementary appendix exhibit A3). In rural and urban areas, non-Hispanic Black individuals were more likely to rely on independently owned pharmacies for access compared with non-Hispanic White individuals (*P* < .001), although the absolute difference was small (supplementary appendix exhibit A3).

**Figure 1.**
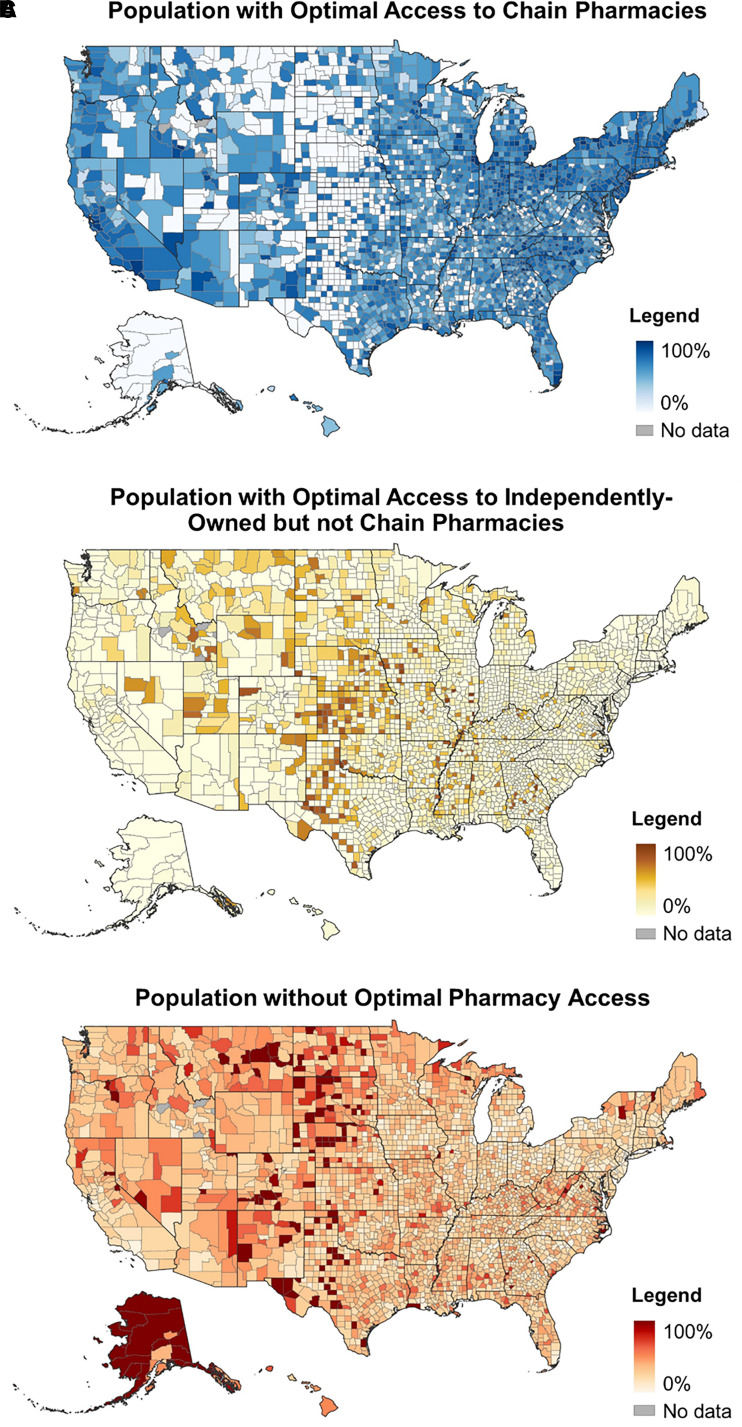
County-level variation in pharmacy access. The maps show the county-level proportion of individuals with optimal access to chain pharmacies (*A*), with optimal access to independently owned but not chain pharmacies (*B*), and without optimal pharmacy access (*C*). Optimal access was defined as driving distance <2 miles in urban counties, <5 miles in suburban counties, and <10 miles in rural counties.

Access to chain and independently owned pharmacies presented strong geographic variation across US counties (Figure 1). Access to chain pharmacies was highest along the coasts. In 726 (23.1%) of US counties, less than 20% of the population had optimal access to chain pharmacies (Figure 1A). These counties were concentrated in Montana, North Dakota, South Dakota, Nebraska, Kansas, Colorado, Georgia, Mississippi, and Missouri. In 438 (13.9%) US counties, at least half of the population relied solely on independently owned pharmacies for access (Figure 1B). Counties with at least half of the population relying on independently owned pharmacies for access were concentrated in Montana, South Dakota, Kansas, Nebraska, Oklahoma, Texas, Iowa, Illinois, Kentucky, Mississippi, Missouri, and Georgia. Finally, there were 392 (12.5%) counties where more than half of the population lacked optimal pharmacy access (Figure 1C); these counties were concentrated in Alaska, Montana, North Dakota, South Dakota, Nebraska, and Texas.

## Discussion

Our study measured pharmacy access at the individual level for a nationally representative sample of the US population. We estimated that 59.0 million US individuals do not have optimal pharmacy access, and that an additional 15.1 million rely on independently owned pharmacies to access pharmacy services. Individuals relying on independently owned pharmacies for access were more likely to live in rural areas, be above sixty-five years of age, and belong to low-income households.

Our findings are consistent with previous reports of the prominent role of independently owned pharmacies in serving rural areas and underrepresented racial/ethnic communities in urban areas.^6,8,9^ Nevertheless, our study is an important contribution to the literature on pharmacy access because it demonstrates the role of independently owned pharmacies in ensuring an equitable access to pharmacy services across income strata and the rural–urban continuum. States with lower rates of optimal pharmacy access coincided with states more heavily relying on independently owned pharmacies for access, which demonstrates that the closures of independently owned pharmacies exacerbate geographic disparities in pharmacy access.

Because independently owned pharmacies handle relatively low script volume compared with chains, they have limited power to negotiate contracts with pharmaceutical benefit managers. As a result, independently owned pharmacies often form part of pharmacy service administrative organizations, which negotiate on behalf of large numbers of independent pharmacies. By aggregating volume, pharmacy service administrative organizations are able to negotiate more favorable contracts with pharmaceutical benefit managers than independently owned pharmacies would negotiate on their own. Four pharmacy service administrative organizations dominate the market and represent over 80% of independently owned pharmacies.^18^ A recent analysis revealed that the 2023 Part D preferred cost-sharing networks of four insurers that account for over half of Part D enrollees do not include any of the leading pharmacy service administrative organizations.^10,18,19^ In other words, the four insurers that control the majority of the Part D market excluded the vast majority of independently owned pharmacies from their preferred cost-sharing networks in the current year. These trends are concerning for two reasons. First, the hardship associated with the exclusion from preferred Part D pharmacy networks puts independently owned pharmacies at increased risk of closure, jeopardizing access not only for Medicare beneficiaries but also for other populations who rely on them for accessing pharmacy services. Second, the exclusion of independently owned pharmacies from preferred cost-sharing networks places additional access barriers for beneficiaries who do not have optimal access to in-network chains, as they face higher out-of-pocket costs if they use their local, independently owned pharmacy. Alternatively, they may face longer driving distances if they use the preferred cost-sharing pharmacy. While there is no access standard for networks of preferred cost-sharing pharmacies (statutory requirements are only established for the entire networks of Part D plans),^14^ future research should evaluate to what extent the exclusion of independently owned pharmacies from preferred cost-sharing networks hinders pharmacy access, particularly for plans serving areas with suboptimal access to chain pharmacies.

Although suboptimal pharmacy access was particularly pronounced in rural areas, 17.1% of urban residents lacked optimal pharmacy access. This finding is important and calls for the development of reimbursement models that not only consider the urbanicity of a pharmacy in the tiering of dispensing fees, as some state Medicaid programs do,^20^ but also the degree of unmet need of an area. Given the considerable financial investment required for the opening of pharmacies, policymakers should prioritize the development of interventions that prevent the closure of pharmacies that are the only source of medications, vaccines, and rapid diagnostic testing in an area.

Some limitations should be considered when interpreting these data. First, our measures of spatial access to care are based on driving distance. Driving time is likely a more valid measure of real-world access burden. Unfortunately, driving distances and times correlate very differently at different settings and scales and computing drive times is technologically and computationally onerous. Second, our estimates do not account for variability in car ownership and availability of public transportation. Third, our measures of access only measure the spatial dimension of pharmacy access and do not consider cost-related barriers to accessing medications and pharmacy services. Fourth, some differences may be statistically significant due to the large sample size available but may not be relevant due to the small effect size. Fifth, our results are based on a synthetic population of the United States and therefore have limited implications for health policy outside of the United States.

Despite these limitations, our individual-level study constitutes a major methodological innovation as it evaluated pharmacy access at the individual level for over 90 million individuals. The use of a 30% random sample of the RTI US Household Synthetic Population enabled the execution of highly accurate spatial analyses for a nationally representative sample of the United States. The sampling was needed due to the large computational power required to execute spatial analyses for large samples; nevertheless, the sampling was performed at the block group level, which ensured that the resulting sample was representative of the US population nationwide. The availability of income and racial/ethnic data for a large sample enabled the detection of inequities in pharmacy access across the rural–urban continuum.

## Conclusion

We measured pharmacy access at the individual level for a nationally representative sample of the US population and found that 59 million US individuals lack optimal pharmacy access. Further, an additional 15 million individuals solely rely on independently owned pharmacies for access. Rural populations, older adults, and low-income households were more likely to rely on independent pharmacies for accessing pharmacy services, which demonstrates the critical role of independently owned pharmacies in ensuring equity in pharmacy access. Our study reveals that the closure of independently owned pharmacies may exacerbate existing inequities in health care access.

## Supplementary material

Supplementary material is available at *Health Affairs Scholar* online.

## Supplementary Material

qxad003_Supplementary_Data

## References

[1] Surya S, Seeley E. Competition, consolidation, and evolution in the pharmacy market. Published August 12, 2021. Accessed January 19, 2023. https://www.commonwealthfund.org/publications/issue-briefs/2021/aug/competition-consolidation-evolution-pharmacy-market

[2] Georgetown University Health Policy Institute. Data profiles: prescription drugs. Published April 30, 2014. Accessed December 13, 2022. https://hpi.georgetown.edu/rxdrugs/

[3] Grabenstein JD. Essential services: quantifying the contributions of America's pharmacists in COVID-19 clinical interventions. J Am Pharm Assoc. 2022;62(6):1929–1945.e1. 10.1016/j.japh.2022.08.010 PMC9387064 36202712

[4] San-Juan-Rodriguez A, Newman TV, Hernandez I, et al Impact of community pharmacist-provided preventive services on clinical, utilization, and economic outcomes: an umbrella review. Prev Med. 2018;115:145–155. 10.1016/j.ypmed.2018.08.029 30145351

[5] Newman TV, San-Juan-Rodriguez A, Parekh N, et al Impact of community pharmacist-led interventions in chronic disease management on clinical, utilization, and economic outcomes: an umbrella review. Res Social Adm Pharm. 2020;16(9):1155–1165. 10.1016/j.sapharm.2019.12.016 31959565

[6] Berenbrok LA, Tang S, Gabriel N, et al Access to community pharmacies: a nationwide geographic information systems cross-sectional analysis. J Am Pharm Assoc. 2022;62(6):1816–1822.e2. 10.1016/j.japh.2022.07.003 35965233

[7] Guadamuz JS, Alexander GC, Zenk SN, Qato DM. Assessment of pharmacy closures in the United States from 2009 through 2015. JAMA Intern Med. 2020;180(1):157–160. 10.1001/jamainternmed.2019.4588 31633745 PMC6806432

[8] Mshca ELD, Fred Ullrich BA, Mueller KJ. Update on rural independently owned pharmacy closures in the United States, 2003–2021. Rural Policy Research Institute Rural Policy Brief. https://rupri.public-health.uiowa.edu/publications/policybriefs/2022/Independent%20Pharmacy%20Closures.pdf

[9] Guadamuz JS, Wilder JR, Mouslim MC, Zenk SN, Alexander GC, Qato DM. Fewer pharmacies in Black and Hispanic/Latino neighborhoods compared with White or diverse neighborhoods, 2007–15. Health Aff (Millwood). 2021;40(5):802–811. 10.1377/hlthaff.2020.01699 33939507

[10] Fein AJ. Small pharmacies walk away from medicare Part D's 2023 preferred networks. Accessed January 19, 2023. https://www.drugchannels.net/2022/12/small-pharmacies-walk-away-from.html

[11] National Council for Prescription Drug Programs. DataQ. Accessed August 28, 2022. http://dataq.ncpdp.org/

[12] Rineer JI, Kery C, Downey TW, et al RTI SynthPopTM U.S. Synthetic Populations 2019. RTI International; 2021.

[13] OpenStreetMap Contributors. Planet dump. Accessed October 24, 2022; 2015. https://planet.openstreetmap.org

[14] Access to covered Part D drugs. 42 CFR 423.120. Accessed January 24, 2023. https://www.govinfo.gov/app/details/CFR-2011-title42-vol3/CFR-2011-title42-vol3-sec423-120

[15] US Census Bureau. 2010 census urban and rural classification and urban area criteria. Accessed October 10, 2022. https://www.census.gov/programs-surveys/geography/guidance/geo-areas/urban-rural/2010-urban-rural.html

[16] Ratcliffe M, Burd C, Holder K, Fields A. Defining rural at the U.S. Census Bureau. https://www2.census.gov/geo/pdfs/reference/ua/Defining_Rural.pdf

[17] US Department of Agriculture. Rural-urban continuum codes. Accessed August 30, 2022. https://www.ers.usda.gov/data-products/rural-urban-continuum-codes.aspx

[18] Fein AJ. McKesson leads another round of PSAO consolidation. Accessed January 20, 2023. https://www.drugchannels.net/2018/04/mckesson-leads-another-round-of-psao.html

[19] Cubanski J, Damico A, Neuman T. Kaiser family foundation issue brief. Medicare Part D in 2018: the latest on enrollment, premiums, and cost sharing. Accessed January 20, 2023. https://www.kff.org/medicare/issue-brief/medicare-part-d-in-2018-the-latest-on-enrollment-premiums-and-cost-sharing/

[20] Medicaid Covered Outpatient Prescription Drug Reimbursement Information by State. Accessed January 24, 2023. https://www.medicaid.gov/medicaid/prescription-drugs/state-prescription-drug-resources/medicaid-covered-outpatient-prescription-drug-reimbursement-information-state/index.html

